# Characterization of PM_2.5_ Carbonaceous Components in a Typical Industrial City in China under Continuous Mitigation Measures

**DOI:** 10.3390/toxics12070461

**Published:** 2024-06-26

**Authors:** Hongya Niu, Chunmiao Wu, Michael Schindler, Luis F. O. Silva, Bojian Ma, Xinyi Ma, Xiaoteng Ji, Yuting Tian, Hao Zhu, Xiaolei Bao, Yanhai Cheng

**Affiliations:** 1 School of Earth Sciences and Engineering, Hebei University of Engineering, Handan 056038, China; niuhongya@hebeu.edu.cn (H.N.); w2410885109@163.com (C.W.); maxinyi19991221@163.com (X.M.); xt727398@163.com (X.J.); m19933132704@163.com (Y.T.); haozhu001110@163.com (H.Z.); 2 Department of Earth Sciences, University of Manitoba, Winnipeg, MB R3T 2N2, Canada; michael.schindler@umanitoba.ca; 3 Department of Civil and Environmental Engineering, Universidad de la Costa, Barranquilla 080002, Colombia; lfsoacademico@gmail.com; 4 Postgraduate Doctoral Program in Society, Nature and Development, Universidade Federal Do Oeste Do Pará, UFOPA, Paraná 68040-255, Brazil; 5 CDLAC—Data Collection Laboratory and Scientific Analysis LTDA, Nova Santa Rita 92480-000, Brazil; 6 Hebei Advanced Environmental Protection Industry Innovation Center Co., Ltd., Shijiazhuang 050026, China; 18660902208@163.com; 7 Hebei Chemical & Pharmaceutical College, Shijiazhuang 050026, China; bxl5@163.com

**Keywords:** carbonaceous components, PM_2.5_, long-term change, source analysis, Handan City

## Abstract

The goals of the “dual carbon” program in China are to implement a series of air pollution policies to reduce the emission of carbon-bearing particulate matter (PM). Following improvements in the reduction in carbon emissions in Handan City, China, fine particulate matter (PM_2.5_) was collected in the winters from 2016 to 2020 to characterize the concentrations and sources of carbonaceous components in PM_2.5_. Trend analysis revealed that both organic carbon (OC) and elemental carbon (EC) concentrations significantly decreased. The proportion of total carbon aerosol (TCA) in PM_2.5_ decreased by 47.0%, highlighting the effective reduction in carbon emissions. Secondary organic carbon (SOC) concentrations increased from 2016 (12.86 ± 14.10 μg·m^−3^) to 2018 (36.76 ± 21.59 μg·m^−3^) and then declined gradually. SOC/OC was larger than 67.0% from 2018 to 2020, implying that more effective synergistic emission reduction measures for carbonaceous aerosol and volatile organic compounds (VOCs) were needed. In the winters from 2016 to 2020, primary organic carbon (POC) concentrations reduced by 76.1% and 87.6% under a light/moderate pollution period (LP) and heavy/severe pollution periods (HPs), respectively. The TCA/PM_2.5_ showed a decreasing trend under LP and HP conditions, decreasing by 42.1% and 54.7%, respectively. Source analysis revealed that carbonaceous components were mainly from biomass burning, coal combustion and automotive exhaust emissions in the winters of 2016 and 2020. OC/EC and K^+^/EC analysis pointed out that air pollutant reduction measurements should focus on rectification biomass fuels in the next stage. Compared with 2016, the contributions of automotive exhaust emissions decreased in 2020. OC and EC concentrations decreased due to control measures on automotive exhaust emissions.

## 1. Introduction

With rapid socioeconomic development and the increase in energy consumption, the problem of air pollution is becoming more serious, and atmospheric particulate matter is one of the major pollutants [1,2]. Carbonaceous components are a fundamental part of fine particulate matter (PM_2.5_) in the atmosphere, typically account for 10–60% of PM_2.5_ mass concentration and can reach up to 90% in some areas, so they are considered a key constituent of air pollution [3,4]. Carbonaceous components are mainly composed of organic carbon (OC) and elemental carbon (EC). OC is of complex origin and is derived from primary organic carbon (POC), directly emitted from pollution sources and secondary organic carbon (SOC) formed by complex photochemical reactions of gaseous precursors, such as O_3_ and volatile organic compounds (VOCs). EC originates mainly from the incomplete combustion of fossil fuels and biomass and can be used as a tracer for primary emissions due to its chemical stability [5,6]. Studies show that 85% of EC and 82% of OC in the atmosphere are predominantly present in PM_2.5_ [7]. OC contains a variety of toxic and carcinogenic substances. Atmospheric OC has the ability to scatter sunlight, whereas atmospheric EC has a high adsorption capacity and a strong absorption effect on both visible and infrared light [8,9]. These properties of atmospheric OC and EC affect visibility and radiation balance [10]. Hence, the effects of carbonaceous PM_2.5_ on air quality and radiation budget requires in-depth studies on its abundance, size distribution and chemical composition.

To achieve an overall improvement in China’s air quality, the State Council promulgated the “Air Pollution Action Plan” and “Blue Sky Action Plan” in 2013 and 2018, respectively, and put forward specific pollutant reduction measures and targeted requirements for different regions. As the main constituent of air pollution, PM_2.5_ is an important target of various pollutant reduction policies. As a consequence of these policies, the annual average concentrations of PM_2.5_ decreased from 72 μg·m^−3^ in 74 key cities in 2013 to 39 μg·m^−3^ in 168 cities in 2020 [11,12]. During this period, PM_2.5_ in the Beijing-Tianjin-Hebei (BTH) and the Yangtze River Delta (YRD) regions decreased by 51.9% and 47.8%, respectively. As an important component in air pollution, the rapid decline in total PM_2.5_ concentrations was accompanied by changes in the industrial structure, composition, size and sources of carbonaceous emissions. In this regard, Ji et al. showed that the concentrations of OC and EC decreased in carbonaceous aerosols in the urban areas of Beijing over five and half years [13]. The authors showed that the ratios of OC and EC in PM_2.5_ mass did not change significantly during this period. Furthermore, Chen et al. identified higher proportions of SOC in the PM_2.5_ of the Chengdu area in 2020 than in 2016 [14]. The authors also showed that the proportions of OC in PM_2.5_ remained at a constant level during periods of high air pollution and that the contributions of secondary organic components to PM_2.5_ were higher in 2020 than 2016. Luo et al. also found that the concentrations of carbon components in PM_2.5_ in Baoding City had decreased continuously since the implementation of clean heating measures, which reduced the contribution of primary emissions (biomass burning and coal combustion) to carbonaceous aerosols [15]. Recently, Chen et al. showed that the carbon fractions in PM_2.5_ of the Nanjing area declined rapidly after the implementation of the Ten Statements of Atmosphere, but the decrease slowed down in the later period and that the proportions of SOC in PM_2.5_ increased rather than decreased [16]. These examples show that changes in the concentration, composition and sources of carbon components in PM_2.5_ need to be monitored for assessing the effectiveness of air quality improvements, and for adjusting and optimizing future pollution prevention and control measures. Long-term field measurements are essential for evaluating the effects of air pollution control strategies. However, studies on long-term changes in the composition of carbon components and their sources in industrial cities of northern China are still lacking. This study analyzes the composition of carbonaceous components in the PM_2.5_ of the Handan area during the winters of 2016 to 2020 and provides direct evidence that the mass concentrations of the carbon components are currently decreasing. 

Handan is an industrial city in the south of Hebei Province and has a large number of steel and cement enterprises. Hebei is part of the BTH region and surrounding areas. It is one of the economic core regions and the most severe air pollution region in China [17]. Emission inventories for OC and EC show that the BTH region and surrounding areas are the regions with the largest carbonaceous aerosol emissions in China [18]. The sources of carbonaceous aerosols are predominantly fuel combustions, such as emissions from automotive exhaust systems and coal combustions [19]. In 2017, ten ministries and commissions issued the “Planning for Clean Heating in Winter in Northern Regions (2017–2021)” to replace private and industrial coal burning with various clean heating methods. These measures played an important role in alleviating air pollution problems in the winter months in northern China, especially the BTH region.

In Handan City, the air pollution was severe. It was consistently ranked among the top-ten polluted cities in terms of air quality according to the Ministry of Ecology and Environment. In recent years, with the implementation of policies, such as the Air Pollution Action Plan and the Blue Sky Action Plan, and the Planning for Clean Heating, the air quality has improved in Handan, and it was subsequently removed from the list of the top-ten polluted cities. This study shows that the sustained improvements in air quality in Handan are accompanied by changes in the composition, concentration and sources of carbon components in PM_2.5_.

## 2. Materials and Methods

### 2.1. Sample Collection

**Figure 1:**
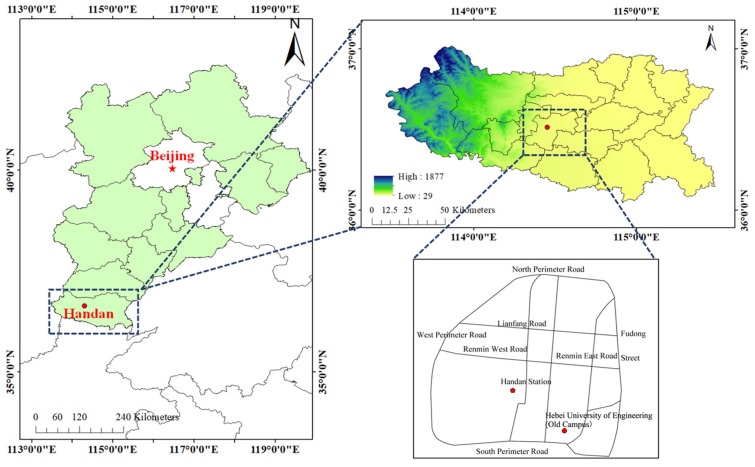
Location of the PM_2.5_ sampling site in Handan City.

The sampling site for PM_2.5_ is located on the roof of the former administrative building of the Hebei University of Engineering (36.57°N, 114.50°E, the national monitoring station of the Handan Mining Institute) and is approximately 16 m above ground level (Figure 1). The sampling site is located in the school area, surrounded by a residential area, with no large buildings or factories in the vicinity. The South Gate is adjacent to the South Ring Road where the traffic flow is high. A large steel plant (Hansteel Company of the HBIS Group), a thermal power plant and a cement plant are located about 5.8 km from the sampling site.

PM_2.5_ samples were collected in Handan City in the winters of 2016–2020 (December to February of the following year). The sampling instrument was a multichannel airborne particulate sampler (UnrayZR-3930D), and the sampling collector was a quartz filter membrane. Two samples were collected every day respectively from 8:00 to 19:30 (daytime) and from 20:00 to 7:30 of the next day (nighttime), with a sampling duration of 11.5 h.

### 2.2. Sample Analysis

The quartz filters were baked in a muffle furnace at 550 °C for 5.5 h before sampling to remove the impact of organic impurities in the filters. Quartz filters were placed in a thermostat (at the temperature of ±25 °C and relative humidity of ±30%) for at least 24 h. Subsequently, the filters were weighed using an electronic balance with a precision of 1/100,000 (10 μg) (XS205 DualRange, Zurich, Switzerland), and the samples were processed and weighed again under the same conditions after the sampling was completed. Finally, the filters were stored in a freezer at temperatures below −18 °C for subsequent chemical analysis.

A Thermal/Optical Carbon Analyzer developed by the Desert Research Institute (DRI model 2001A) of the United States was used for the analysis of the carbon components. The instrument utilized the thermal/optical reflectance (TOR) method of the IMPROVE (Interagency Monitoring of Protected Visual Environments) protocol for detection [20]. The lowest detection limit for OC and EC is 0.2 μg·cm^−2^. Quartz filter membranes were placed in pure helium environments at 120 °C (OC1), 250 °C (OC2), 450 °C (OC3) and 550 °C (OC4), respectively, to convert the particulate carbon on the filter membrane into CO_2_. The samples were then placed in a mixture of 2% O_2_ and 98% He, and heated at 550 °C (EC1), 700 °C (EC2) and 800 °C (EC3), respectively, at which time the elemental carbon in the samples was released. The CO_2_ generated in the above temperature gradients was catalyzed by MnO_2_ and converted to CH_4_ in a reducing environment, which could be detected by a flame ionization detector (FID). During heating of the sample, part of the organic carbon can be pyrolyzed to form optical pyrolytic carbon (OPC). According to the IMPROVE protocol: OC = OC1 + OC2 + OC3 + OC4 + OPC; EC = EC1 + EC2 + EC3-OPC.

Water soluble ion analysis uses an ion chromatograph (Thermo, Dionex, ICS-600, California, USA). The quartz filter membrane with a radius of 8 mm was put into the centrifuge tube, and 20 mL ultra-pure water was added into the centrifuge tube to immerse the filter membrane. The filter was subsequently put into an ultrasonic bath (with an ice pack) to dissolve ions under ultrasonic conditions. After 30 min, the ice pack was replaced, and the ultrasonic treatment was continued for 30 min. After 5 min settling, the solution was then filtered through a 0.22 μm microporous water filter. Finally, the sample was placed into an ion chromatography automatic injector to determine the concentrations of the cations.

### 2.3. Estimation of SOC Mass Concentration

This study uses the EC tracer method to calculate the concentrations of SOC [21]. The basic assumption is that the EC in atmospheric particulates is chemically stable, originates mainly from primary emission sources and correlates well with the amount of directly emitted POC. The empirical equation is as follows:

(1) $$\mathrm{POC}=\mathrm{EC}\times {\left(\mathrm{OC}/\mathrm{EC}\right)}_{\mathrm{pri}}+N$$

**Figure 2:**
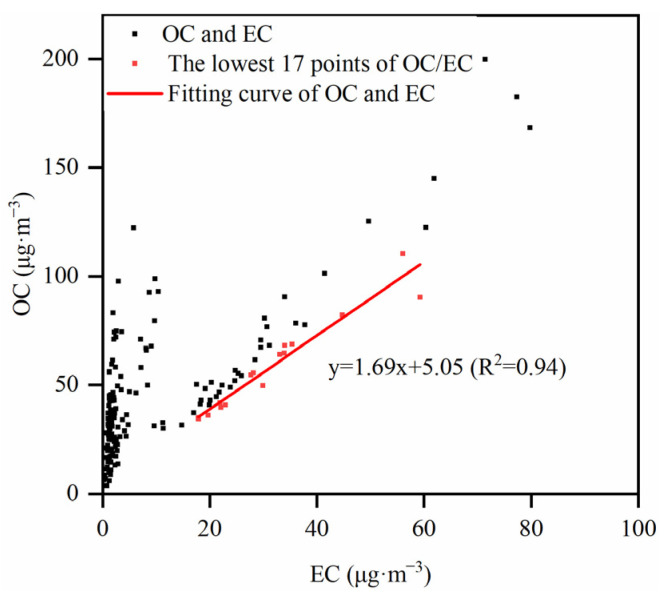
Linear regression of OC and EC from 2016 to 2020 in winter.

(2) SOC=OC−POC

where (OC/EC)_pri_ represents the ratio of OC/EC emitted by primary combustion sources, and N represents organic carbon emitted by primary noncombustion sources (e.g., road dust or biogenic emissions). According to Lim et al., more accurate results can be obtained by using 5% to 10% of the samples (with the total number of samples larger than 20) and the smallest OC/EC values for the estimation of (OC/EC)_pri_ [21]. In this study, the top 10% of the smallest OC/EC values of all samples were selected from 2016 to 2020, and then OC and EC mass concentration values were analyzed by linear regression analysis (Figure 2). The slope of the regression equation is (OC/EC)_pri_, and the intercept value is N. The R^2^ of 0.94 indicates a strong linear dependence. We assumed that 17 samples were generated from primary emissions and were not subjected to mixing in the atmosphere. The slope and intercept of the fitted curves were inserted into Equations (1) and (2) for the calculation of SOC.

### 2.4. Principal Component Analysis (PCA)

Eight carbon components of PM_2.5_ sampled in the winters of 2016 and 2020 were analyzed by PCA (using SPSS 19.0 software). The KMO metric (winter 2016: 0.831; winter 2020: 0.643) and Bartlett’s test of sphericity (*p* < 0.05) showed that the correlation between the data was good, so that the carbon components of PM_2.5_ were suitable for the principal component analysis. The factors with eigenvalues greater than 1 were extracted as principal factors. The rotated component matrix can express the coefficients of the variables in each factor, i.e., the loadings of the variables in the factors. In this study, the orthogonal rotation method was used to rotate the component matrix, and components with loadings greater than 0.8 were considered as factors with close associations to pollution sources.

## 3. Results and Discussion

### 3.1. Characteristics of PM_2.5_ and Carbonaceous Components 

#### 3.1.1. Variation Trends during the Winters

**Figure 3:**
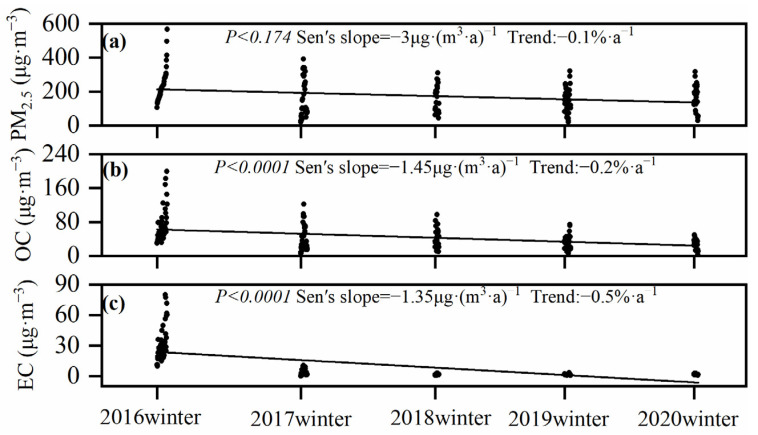
Variation trends of PM_2.5_, OC and EC concentrations in the winters from 2016 to 2020 in Handan City: (**a**) PM_2.5_ variation trend; (**b**) OC variation trend; (**c**) EC variation trend.

According to the Mann–Kendall test and Sen’s slope analysis (Figure 3), the concentrations of OC and EC in PM_2.5_ significantly decreased during the monitored period [OC: *p* < 0.0001, −1.45 μg·(m^3^·a)^−1^, −0.2%·a^−1^; EC: *p* < 0.0001, −1.35 μg·(m^3^·a)^−1^, −0.5%·a^−1^]. The EC values originated from primary pollutant emissions and their decrease in concentration was more significant than for OC. This decrease indicates a reduction in primary emission sources in Handan City as a consequence of the implemented air pollution reduction policies, which focused on a reduction in coal furnaces and stricter vehicle pollution controls during mitigation actions. For example, Handan City has eliminated more than 178,000 old motor vehicles since 2013, and Hebei Province carried out a special action of “boiler removal and chimney demolition” in 2014. It eliminated a total of 8651 coal-fired boilers (12,965 steam tons) and zeroed out coal-fired boilers below 35 steam tons. Since the implementation of the “double replacement” project in 2015, a total of 1,519,800 households have been renovated for clean heating. Fang et al. found that coal combustion emissions were the primary source of EC in the BTH region (50% on average in 2012–2013) [22]. Hence, the more significant decrease in EC relative to OC in Handan City indicates a large reduction in carbonaceous components emitted from coal combustion. Compared with OC and EC, the decreasing trend of PM_2.5_ was not significant [*p* < 0.174, −3 μg·(m^3^·a)^−1^, −0.1%·a^−1^], indicating that air pollution reduction strategies had a limited improvement on PM_2.5_ emissions. While the concentrations of carbonaceous components were substantially reduced under heavy pollution conditions during the winter months, the reduction in the concentration of PM_2.5_ is still a crucial problem and requires further actions with respect to emission reductions and the monitoring of emissions from targeted sources.

#### 3.1.2. Mass Concentration and Percentage Variation

**Figure 4:**
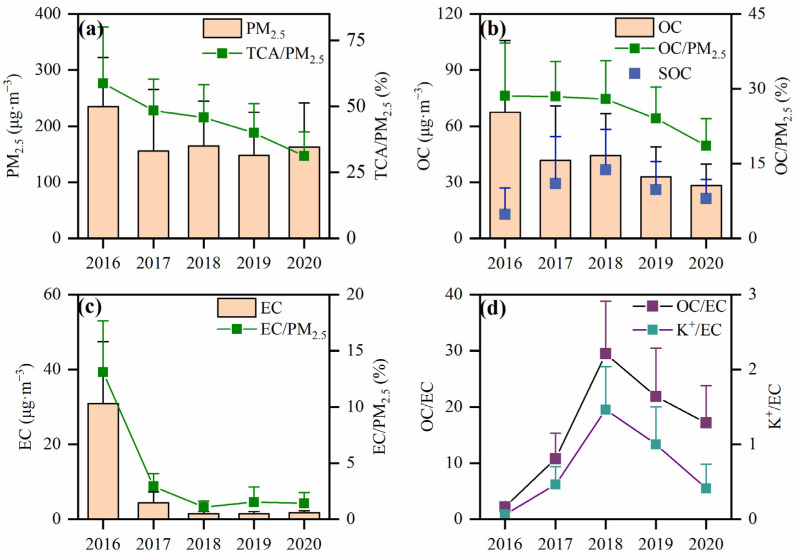
Concentrations and percentage distributions of PM_2.5_, OC, EC and K^+^ in the winters from 2016 to 2020: (**a**) PM_2.5_ concentration and proportion of TCA in PM_2.5_; (**b**) concentrations of OC and SOC and proportion of OC in PM_2.5_; (**c**) EC concentration and proportion of EC in PM_2.5_; (**d**) value of OC/EC and value of K^+^/EC (whiskers represent the standard error).

The daily mean PM_2.5_ concentrations in the winters from 2016 to 2020 were 235 ± 88 μg·m^−3^, 156 ± 109 μg·m^−3^, 165 ± 80 μg·m^−3^, 148 ± 77 μg·m^−3^ and 163 ± 79 μg·m^−3^ respectively, among which the PM_2.5_ concentration remained at a relatively stable level from 2017 to 2020 (Figure 4). However, the PM_2.5_ concentrations in Handan City during the sampling period all significantly exceeded the daily average standard (75 μg·m^−3^) of Class II of China’s Ambient Air Quality Standards (GB 3095-2012) issued in February, 2012 [23]. The concentrations of OC and EC reduced significantly: OC concentrations decreased in 2016–2017, rose again in 2018 and continued to decrease in 2019–2020; EC concentrations decreased in 2016–2018 and stabilized in 2019–2020. In 2017, the average concentrations of PM_2.5_, OC and EC decreased by 33.5%, 38.1%, 85.6%. In comparison to the observations in 2013 (PM_2.5_: 239 μg·m^−3^; OC: 32.4 μg·m^−3^; EC:14.4 μg·m^−3^) [24], the decrease in the concentrations of these pollutants can be attributed to the implementation of large-scale pollution control measures and the cumulative effect of long-term mitigation in Handan City. To gain a better understanding of the relative contributions of OC and EC to PM_2.5,_ it is important to develop effective air pollutant reduction measures. Over the five years, the total proportions decreased with OC contributing higher proportions to PM_2.5_ (2016: 28.6% ± 10.6%, 2017: 28.5% ± 7.0%, 2018: 27.9% ± 7.7%, 2019: 24.0% ± 6.3% and 2020: 18.6% ± 5.4%) than EC (2016: 13.1% ± 4.6%, 2017: 2.9% ± 1.1%, 2018: 1.1% ± 0.6%, 2019: 1.5% ± 1.4% and 2020: 1.4% ± 1.0%). 

The total carbon aerosol (TCA) value represents the sum of all organic matter (OM) and elemental carbon in PM_2.5_. According to Turpin et al., the average molecular weight per carbon weight is 1.6 ± 0.2 for urban aerosols and follows the equation TCA = 1.6 × OC + EC [25]. Over the five years, the average concentrations of TCA in the winters were 138.8 ± 77.6 μg·m^−3^, 71.1 ± 49.2 μg·m^−3^, 72.4 ± 36.2 μg·m^−3^, 54.2 ± 26.1 μg·m^−3^ and 46.9 ± 18.6 μg·m^−3^, respectively, and the respective values for TCA/PM_2.5_ were 58.8% ± 21.3%, 48.4% ± 11.9%, 45.8% ± 12.5%, 34.0% ± 11.0% and 31.2% ± 9.1%, respectively. The values of TCA/PM_2.5_ were higher than 20% over this time span, indicating that carbonaceous aerosols were crucial component of PM_2.5_ (Figure 4). However, the respective TCA/PM_2.5_ decreased by 47.0%, and this was closely related to a series of air pollution prevention and control measures taken by Handan City in recent years.

Table 1 lists the recently published concentrations of OC and EC in the main domestic and foreign cities. Although the monitoring periods differ, a comparison of the concentrations of the carbonaceous component between different cities can reveal important information for policymakers with respect to energy consumptions and air quality improvements in other countries [26]. Here, OC and EC concentration levels in Handan City are significantly higher than those in other developed-country cities, such as Tokyo in Japan, Seoul in South Korea, Paris in France and Athens in Greece. The reason for this is the proportional higher use of clean energy and the adoption of more stringent air pollution reduction measures in the latter countries. In comparison to domestic cities, EC concentrations in Handan City are lower than those of Xi’an, Harbin and Lanzhou, but close to Guangzhou. Furthermore, OC concentrations in Handan City are lower than those of Xi’an and Baoding, and significantly higher than Guangzhou and Lhasa. These differences and similarities indicate that in comparison to other domestic cities, the OC and EC concentrations in Handan City improved, especially with respect to the latter concentrations. However, in order to further improve the quality of the atmospheric environment, more effective synergistic carbonaceous aerosol and VOC reduction measures need to be taken in the context of the “dual carbon” program.

#### 3.1.3. Correlation Analysis and SOC Variation Characteristics

**Figure 5:**
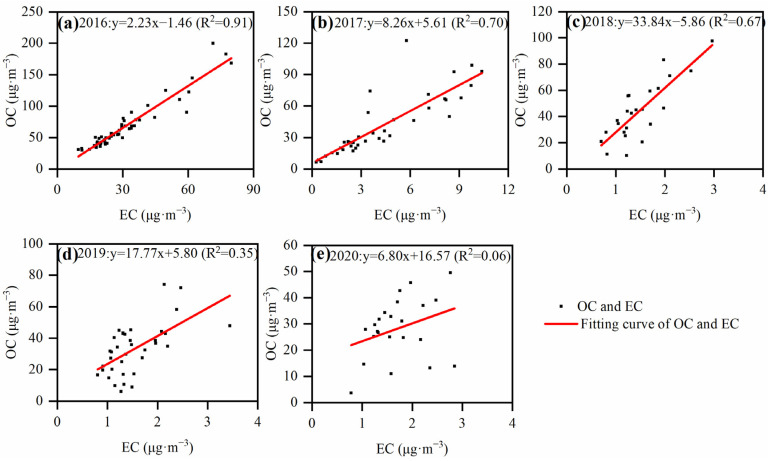
Correlation between OC and EC in the winter: (**a**) 2016; (**b**) 2017; (**c**) 2018; (**d**) 2019; (**e**) 2020.

Correlations between OC and EC can be used to preliminarily investigate the homology of OC and EC, and to identify a common source of both components, i.e., the higher the correlation coefficient, the higher the possibility of the two having the same source [26]. For Handan City, correlation coefficients show a gradual decreasing trend from 2016 to 2020 (2016: R^2^ = 0.91, 2017: R^2^ = 0.70, 2018: R^2^ = 0.67, 2019: R^2^ = 0.35, 2020: R^2^ = 0.06; Figure 5), indicating a gradual deterioration of source consistency between OC and EC. It shows furthermore that OC and EC originated in the early recording period from similar sources, mainly coal combustion and vehicle exhausts. With the implementation of various carbon reduction emission measures, such as the conversion of coal to biomass, it led to a more dispersed source of carbon components than in the early period. Ratios of OC to EC can be used to identify various pollution sources. For example, OC/EC ratios between 1.0 and 4.2, 2.5 and 10.5 and 16.8 and 40.0 indicate that the predominant sources are automotive exhaust, coal combustion and biomass burning emissions, respectively [37,38,39]. As the OC/EC changed from 2.20 ± 0.33 (2016) to 10.76 ± 4.57 (2017), 29.47 ± 9.34 (2018), 21.88 ± 8.61 (2019) and 17.14 ± 6.66 (2020), the sources of carbonaceous aerosols gradually shifted from automotive exhaust emissions to biomass burning emissions.

The K^+^/EC ratio can be used to determine the contribution of biomass burning emissions toward EC. Studies have showed that K^+^/EC ratios in fossil and biomass fuels ranged from 0.03 to 0.09 and 0.21 to 0.46, respectively [40,41]. For Handan City, mean K^+^/EC values from 2016 to 2020 were 0.07 ± 0.03, 0.46 ± 0.24, 1.46 ± 0.58, 1.00 ± 0.50 and 0.41 ± 0.32, respectively (Figure 4d). This indicates that biomass burning emissions contributed larger proportions to carbonaceous aerosols in recent winters.

**Figure 6:**
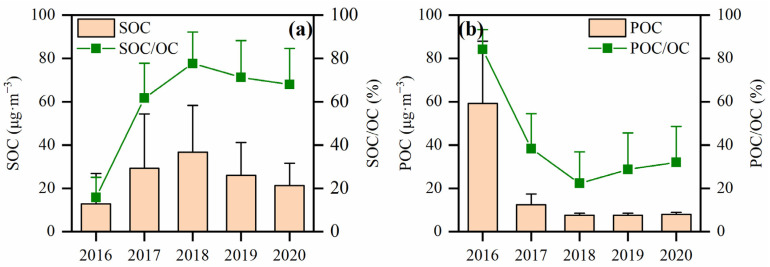
Concentrations and percentage distributions of SOC and POC in the winters from 2016 to 2020: (**a**) SOC concentration and proportion of SOC in OC; (**b**) POC concentration and proportion of POC in OC (whiskers represent the standard error).

The ratio of OC and EC can be also used to determine whether SOC is generated. Chow et al. concluded that SOC exists in the atmosphere when the OC/EC value is greater than 2 [42]. For Handan City, SOC concentrations were 12.86 ± 14.10 μg·m^−3^, 29.29 ± 25.07 μg·m^−3^, 36.76 ± 21.59 μg·m^−3^, 26.03 ± 15.11 μg·m^−3^ and 21.32 ± 10.30 μg·m^−3^ from 2016 to 2020, respectively (Figure 6a). Although the SOC concentrations gradually decreased from 2018 to 2020, they were still higher than in Changchun [43], Chongqing [44], Tianjin [45] and other cities. Over the same time span, the POC concentrations (2016: 59.20 ± 28.80 μg·m^−3^; 2017: 12.44 ± 5.03 μg·m^−3^; 2018: 7.56 ± 0.92 μg·m^−3^; 2019: 7.64 ± 0.93 μg·m^−3^; 2020: 8.01 ± 0.89 μg·m^−3^) (Figure 6b) depict a different trend than the SOC concentrations. However, the ratios SOC/OC and POC/OC stayed relatively stable from 2018 to 2020 and accounted for 67.9–77.6% and 22.4–32.1%, respectively. In comparison to other industrial centers in China such as Langfang (37%) [46] and Shahe (27.6%) [47], the ratios of SOC/OC remained at a high level in Handan City from 2016 to 2020. 

For Handan City, OC and EC concentrations decreased by 38.1% and 85.9% in 2020 compared to 2016. But, during the same time span, the increase in the concentrations of SOC (by 65.8%) and the ratio of SOC/OC indicate increasing contributions of secondary organic components in the carbonaceous components. As SOC is mainly formed from VOCs through a series of complex gas–particle photochemical reactions in the atmosphere, subsequent pollution controls should focus on the reduction of VOCs [44].

### 3.2. Pollution Characteristics of PM_2.5_ and Carbonaceous Components at Different Pollution Levels 

#### 3.2.1. Concentration Variations

**Figure 7:**
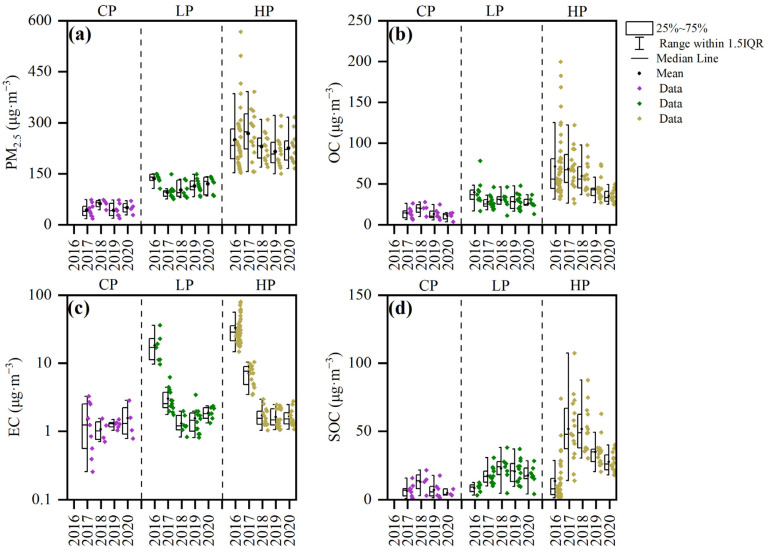
Box plots of PM_2.5_, OC, EC and SOC concentrations at different pollution levels from 2016 to 2020: (**a**) PM_2.5_ concentration; (**b**) OC concentration; (**c**) EC concentration; (**d**) SOC concentration.

Different meteorological conditions and source emission characteristics can lead to differences in the composition of carbonaceous components between polluted days and clean days. Therefore, according to the China’s Ambient Air Quality Standard (GB 3095-2012), in combination with the atmospheric pollution levels of the study area, a clean period (CP) is defined by a daily average PM_2.5_ concentration below 75 μg·m^−3^, a light/moderate pollution period (LP) by a daily average of 75–150 μg·m^−3^ and a heavy/severe pollution period (HP) by a daily average of 150–300 μg·m^−3^. For the sample period 2016–2020, the exclusive occurrences of LP and HP periods during the winter of 2016 resulted in missing CP sample data for this period. In terms of concentrations (Figure 7), PM_2.5_ concentrations did not show any large variations under CP conditions. Under LP conditions, PM_2.5_ concentration decreased from 136 ± 15 μg·m^−3^ in 2016 to 96 ± 19 μg·m^−3^ in 2017 and then increased slowly afterwards. Under HP conditions, the change in PM_2.5_ concentration showed the opposite trend to those of LP, with PM_2.5_ concentration increasing from 250 ± 85 μg·m^−3^ in 2016 to 268 ± 69 μg·m^−3^ in 2017 and then declining with sporadic fluctuations. This opposite trend is a result of a reduction in the local air pollution (caused by a series of environmental protection policies), which resulted in a lower frequency of high PM_2.5_ emission events and thus a decrease and increase in the number of HP and CP/LP periods, respectively. Furthermore, EC concentrations decreased significantly from 2016 to 2017 and changed little from 2018 to 2020. This trend can be explained again by the implementation of a series of pollution reduction policies, which greatly influenced the concentrations of carbonaceous components under HP conditions.

The meteorological conditions of low wind speed and high relative humidity in haze-polluted days prolonged the retention time of precursors in the atmosphere, providing conditions for secondary reactions (Table 2). The combined effect of emissions and meteorology has resulted in high mass concentrations of SOC [48]. For Handan City, SOC concentrations did not show any trend under CP conditions during 2016–2020. However, the SOC concentrations increased and then gradually stabilized under LP and HP conditions. Contrary to the SOC concentrations, the POC concentrations decreased by 76.1% and then gradually stabilized to 87.6% during the five-year span. The latter trend also reflects the implementation of mitigation measures, which controlled primary emissions, namely, coal combustion emissions, biomass burning emissions and automotive exhaust emissions [15].

#### 3.2.2. Percentage Variations

**Figure 8:**
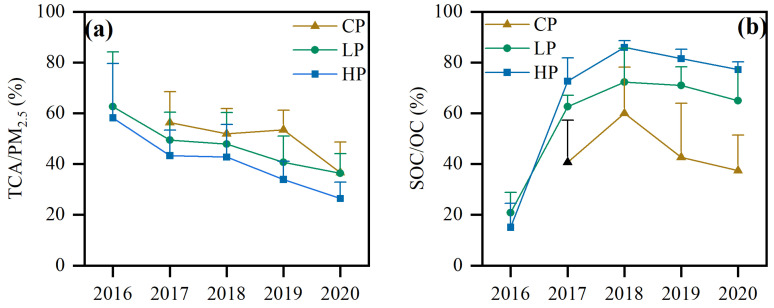
Percentage distributions of TCA/PM_2.5_ and SOC/OC at different pollution levels from 2016 to 2020: (**a**) percentage of TCA/PM_2.5_; (**b**) percentage of SOC/OC (whiskers represent the standard error).

In the time span of 2016–2020, the TCA/PM_2.5_ ratios decreased by 42.1% and 54.7% under LP and HP conditions, respectively (Figure 8). Under CP conditions, the TCA/PM_2.5_ ratio initially declined in 2017–2018, then increased slightly in 2019 and then declined again in 2019–2020. Overall, TCA/PM_2.5_ values showed a decreasing trend under different pollution conditions. With an increase in pollution levels, the concentrations of PM_2.5_ and carbonaceous components continued to increase, but TCA/PM_2.5_ values continued to decrease, which was consistent with the research results of Beijing and Chongqing China [13,44]. This indicates that the increase in other components of PM_2.5_ was higher than the decrease in carbonaceous components. Under HP conditions, both PM_2.5_ and carbon components present decreasing trends in the winters from 2016 to 2020 in Handan City, but the decline in PM_2.5_ concentrations is not as significant as the decline in OC and EC.

Although SOC concentration under HP conditions was higher than that under LP conditions in 2016, SOC/OC values were relatively low, probably due to high concentrations of POC involved in photochemical reactions under HP conditions, resulting in high absolute concentrations of SOC. However, as the increase in OC and SOC were 67.0% and 58.3%, respectively, during the transition from LP to HP, the increase in OC was higher than SOC, resulting in the lowest relative concentrations of SOC calculated from the ratio of the two and the relatively low mass fractions of SOC/OC under HP conditions in 2016. From 2017 to 2020, SOC/OC values increased with pollution levels, indicating that the transformation of SOC was being enhanced with the aggravation of pollution. The SOC/OC stayed high from 2017 to 2020, and were 61.6% ± 16.1%, 77.6% ± 14.5%, 71.3% ± 16.9% and 67.9% ± 16.6%, respectively, suggesting that continuously reducing precursor emissions and then cutting secondary organic transformations were the key to controlling PM_25_ concentrations in Handan [14,16].

### 3.3. Source Analysis

Changes in the sources of the emitted carbonaceous components can be determined using the relative abundances of the constituents during the winters from 2016 and 2020. Studies showed that OC1 originated mainly from biomass burning, OC2 from coal combustions and OC3 and OC4 from road dust or gasoline exhaust. Similarly, OPC is predominantly emitted from biomass burning or gasoline exhaust, EC1 from gasoline exhaust and EC2 and EC3 from diesel exhaust [20,49,50].

Table 3 shows the concentrations of eight carbonaceous components in the winters of 2016 and 2020. Among them, OC2, OC3 and EC1 showed the greatest decrease, indicating the reduced contribution of coal combustion emissions and gasoline exhaust emissions to particulate matter. The concentrations of carbonaceous components in the winter of 2016 were ranked as follows: EC1 > OC1 > OC3 > OC2 > OC4 > OPC > EC2 > EC3. EC1 had the highest concentration, and the concentrations of OC1, OC2 and OC3 did not differ much, indicating that gasoline exhaust, biomass burning and coal combustion emissions were the main sources of carbonaceous components in Handan City in the winter of 2016. The concentrations of carbonaceous components in the winter of 2020 were ranked as follows: OC1 > OPC > EC1 > OC4 > OC2 > OC3 > EC2 > EC3. The concentrations of OC1, OPC and EC1 were relatively high, indicating that biomass burning emissions and gasoline exhaust were the main sources of carbonaceous components in Handan City in the winter of 2020. The concentrations of EC1 and OC1 were both relatively high among the eight components in 2016 and 2020, suggesting that automotive exhaust and biomass burning emission sources contributed greatly to carbonaceous aerosol in Handan City in the winters of 2016 and 2020. In 2016, the concentrations of EC1 and OC1 were the highest and second highest among carbonaceous components, respectively. In 2020, the concentration of OC1 was the highest among carbonaceous components, and the concentration of EC1 dropped to the third rank. The former indicates an increase in the contribution of biomass burning emissions to carbonaceous components and the latter a decrease in the contribution of automotive exhaust in Handan City from 2016 to 2020. These results are consistent with the observed changes in the OC/EC ratios.

PCA is one of the most widely used methods for conducting pollutant source analysis studies. It is a method that reduces the dimensions of data and qualitatively explains the source of components based on their combinatorial characteristics and correlations [51]. A more detailed analysis of changes in the sources of carbonaceous aerosol in Handan City between 2016 and 2020 can be obtained using PCA. During the winter of 2016, OC1, OC2, OC3, EC2 and EC3 had the highest loadings for Factor 1, which explains 61.7% of the total variance, and thus indicates large contributions of combustion and automotive exhaust. OC4 had the highest loadings for Factor 2, which explains 34.0% of the total variance and indicates a high contribution of road dust or automotive exhaust. In the winter of 2020, OC1, EC1, EC2, EC3 and OPC had relatively high loading for Factor 1, which explains 55.1% of the total variance, representing the effects of combustion and automotive exhaust. OC3 and OC4 had the highest loadings for Factor 2, which explains 28.7% of the total variance, representing the influence of road dust or automotive exhaust. These results indicate that the carbonaceous components in the winters of 2016 and 2020 were mainly from biomass burning, coal combustion and automotive exhaust. However, the contribution of Factor 1 in 2016 is higher than its contribution in 2020. Closer inspection of Table 4 shows that OC3 in Factor 1 decreased significantly, with the loadings decreasing from 0.826 to 0.385, so it can be inferred that the decreases in the concentrations of OC and EC can be attributed to the strict control of automobile exhaust emissions.

## 4. Conclusions

Based on the continuous improvement of air quality, PM_2.5_ samples were collected continuously in Handan City in the winters from 2016 to 2020. The OC and EC concentrations were measured with a thermal/optical method, and POC and SOC were quantified with the EC tracer method. According to the Mann–Kendall test and Sen’s slope analysis, both OC and EC showed a significant decrease. TCA/PM_2.5_ decreased by 47.0%, highlighting the effective reduction in carbon emissions. SOC concentrations increased from 2016 to 2018 and decreased continuously after that. However, SOC/OC (67.9–77.6%) maintained a high proportion from 2018 to 2020, which implied that more effective synergistic emission reduction measures were needed for carbonaceous aerosol and VOCs. OC/EC and K^+^/EC analysis pointed out that air pollutant reduction work should focus on rectification biomass fuels in the next stage. From 2016 to 2020, OC concentrations showed a gradual decreasing trend of 101.4% under HP conditions. POC concentrations reduced by 76.1% and 87.6% under LP and HP conditions, reflecting the obvious effect of controlling primary emission sources during periods of high pollution. TCA/PM_2.5_ showed a decreasing trend under LP and HP conditions, decreasing by 42.1% and 54.7%, respectively. 

Source analysis revealed that carbonaceous components were mainly from biomass burning, coal combustion and automotive exhaust emissions in the winters of 2016 and 2020. Due to the control measures on automotive exhaust emissions, OC and EC concentrations decreased, and the contributions of automotive exhaust emissions to carbonaceous components decreased between 2016 and 2020 also. 

These findings demonstrate the effectiveness of the pollution management of carbonaceous components under continuous PM_2.5_ improvements in a typical industrial city. The results also emphasize that carrying out in-depth synergistic emission reduction measures of carbonaceous aerosols and VOCs while continuing to implement air pollution reduction measures are necessary in the future.

## 5. Outlook

This study focused on the characteristics of PM_2.5_ and carbon components in the Handan area over five winter seasons. In the future, it is necessary to characterize the abundance of its components over the entire year. This will allow a better evaluation of the effectiveness of implementations and sustainable mitigation measures, which will provide a more comprehensive scientific basis for regional atmospheric control measures.

## Figures and Tables

**Table 1:** Mass concentrations of OC and EC in main domestic and foreign cities.

Date	Site	OC (μg·m^−3^)	EC (μg·m^−3^)	Reference
From 3 December to 14 December 2020	Handan, China	28.21	1.71	This study
From 27 July to 15 August 2014	Tokyo, Japan	2.2	0.6	[27]
From 11 September 2009 to 10 September 2010	Paris, France	3.0	1.4	[28]
Winter 2020	Seoul, Korea	7.12 ± 2.21	0.70 ± 0.14	[29]
2011–2012	Athens, Greece	5.98	0.92	[30]
Winter 2012–2021	Delhi, India	20.5 ± 11.1	9.4 ± 5.1	[31]
From 1 January to 31 January 2019	Lanzhou, China	14.47 ± 4.58	4.21 ± 1.22	[32]
December, January and February 2017	Xi’an, China	29.02 ± 14.01	6.96 ± 4.16	[33]
From October 2015 to March 2016	Harbin, China	23.50	5.82	[34]
January 2019	Guangzhou, China	6.09 ± 2.64	1.70 ± 1.20	[35]
From May 2013 to March 2014	Lhasa, China	3.27	2.24	[36]
From 1 February to 9 March 2019	Baoding, China	36.63	6.07	[15]

**Table 2:** Meteorological elements at different pollution levels in the winters.

Meteorological Elements	Pollution Levels	2016	2017	2018	2019	2020
Temperature (°C)	CP	-	−2.34	−3.00	−0.83	−1.45
	LP	1.50	−4.47	−2.45	−1.60	0.37
	HP	0.31	1.29	−0.52	0.12	2.47
Relative Humidity (%)	CP	-	34.00	29.00	33.00	37.00
	LP	62.33	45.88	33.50	56.00	64.67
	HP	82.68	58.00	59.00	66.60	72.00
Wind Speed (m·s^−1^)	CP	-	4.46	2.55	2.88	2.90
	LP	3.07	2.77	1.83	1.70	1.40
	HP	2.37	2.83	1.90	2.12	1.24

**Table 3:** Concentrations of eight carbonaceous components in the winters of 2016 and 2020 (μg·m^−3^).

	OC1	OC2	OC3	OC4	EC1	EC2	EC3	OPC
2016	15.24	14.38	14.43	9.29	37.03	0.81	0.36	8.09
2020	8.87	3.92	2.21	5.96	7.09	1.36	0.59	7.39

**Table 4:** Results of the carbon component factor analysis.

Components	Winter of 2016		Winter of 2020	
	Factor 1	Factor 2	Factor 1	Factor 2
OC1	0.938	0.266	0.876	0.333
OC2	0.812	0.569	0.760	0.208
OC3	0.826	0.531	0.385	0.876
OC4	0.075	0.973	0.226	0.946
EC1	0.692	0.711	0.889	0.379
EC2	0.944	0.150	0.803	0.434
EC3	0.948	0.206	0.808	0.147
OPC	0.665	0.725	0.882	0.360
Eigenvalue	4.938	2.719	4.409	2.298
Variance contribution ratio %	61.7	34.0	55.1	28.7
Cumulative variance contribution rate %	61.7	95.7	55.1	83.8

## Data Availability

Data are contained within the article.
